# Herbicidal Activity of the Invasive Weed *Malachra capitata* L.: Growth Stage Dependence, Bioassay-Guided Fractionation, and Physiological Effects on Seed Germination

**DOI:** 10.3390/plants15050832

**Published:** 2026-03-08

**Authors:** Pattharin Wichittrakarn, Sirichai Sathuwijarn, Nutcha Manichart, Kaori Yoneyama, Potjana Sikhao, Naphat Somala, Chamroon Laosinwattana

**Affiliations:** 1 International Academy of Aviation Industry, King Mongkut’s Institute of Technology Ladkrabang, Bangkok 10520, Thailand; pattharin.wi@kmitl.ac.th; 2 Office of Administrative Interdisciplinary Program on Agricultural Technology, School of Agricultural Technology, King Mongkut’s Institute of Technology Ladkrabang, Bangkok 10520, Thailand; sirichai64@yahoo.com (S.S.); potjana.si@kmitl.ac.th (P.S.); naphat.so@kmitl.ac.th (N.S.); chamroon.la@kmitl.ac.th (C.L.); 3 Department of Biochemistry and Molecular Biology, Saitama University, Saitama 338-8570, Japan; yoneyamak@mail.saitama-u.ac.jp

**Keywords:** allelopathy, acid–base solvent partition, natural herbicides, weed control

## Abstract

The invasive weed *Malachra capitata* is unsuitable for human or animal consumption but has recently attracted attention for potential alternative uses. In this study, the allelopathic potential of *M. capitata* for weed control was investigated, as were its allelopathic effects on selected crops. The influence of plant developmental stage on its phytotoxic activity was also assessed. In addition, the physiological effects of the extract on seed germination were investigated. Aqueous leaf extracts were obtained across a range of growth stages and evaluated using seed germination and seedling growth bioassays, followed by bioassay-guided fractionation and GC-MS analysis. Leaves extracts collected at 35 days after planting exhibited the strongest inhibitory activity. Dicot plant species (*Phaseolus lathyroides*, *Cucumis sativus*, *Brassica oleracea*, and *B. chinensis*) were more susceptible to *M. capitata* extracts than grassy species (*Echinochloa crus-galli*, *Zea mays*, and *Oryza sativa*), indicating selective phytotoxicity. In pot experiments, application of leaf residues as surface mulch at rates of 100, 200, and 400 g/m^2^ significantly reduced *P. lathyroides* emergence by 11.25%, 35.00%, and 71.25%, respectively. Bioassay-guided fractionation indicated the ethyl acetate-soluble acidic fraction to contain the active allelochemicals. This inhibition was associated with reduced water uptake and suppression of α-amylase activity during seed germination. The most abundant GC-MS detectable components of the acidic fraction were octadecane (12.45%), eicosane (9.74%), and hexadecane (9.60%). Overall, these findings highlight the allelopathic potential of *M. capitata*, providing a foundation for further applied research and supporting its valorization for sustainable weed management.

## 1. Introduction

**Figure 1:**
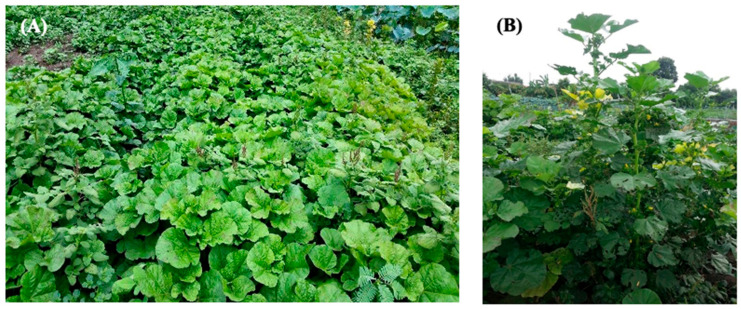
*M. capitata* growing in an experimental field at King Mongkut’s Institute of Technology Ladkrabang (**A**) and morphological representation of the whole plant (**B**).

Chemical herbicides have long been widely used for weed control in agricultural systems due to their effectiveness and ease of application. However, increasing environmental concerns and regulatory restrictions are now driving interest in alternative weed management strategies, including exploration of allelochemicals for the development of natural herbicides [1,2]. Allelopathy is a well-established biological phenomenon in which plants release allelochemicals that influence the growth and development of neighboring plants [3]. Its application in weed management thus offers a potentially cost-effective and environmentally friendly alternative to synthetic herbicides [4,5]. Collectively, allelochemicals comprise a diverse group of secondary metabolites, including phenolics, alkaloids, flavonoids, terpenoids, hydroxamic acids, and glucosinolates [6]. Their phytotoxic effects depend on a number of factors, such as chemical concentration, plant part, developmental stage, and environmental conditions [7]. *Malachra capitata* L. (Malvaceae) is an herbaceous species characterized by coarse hairs, spines, lobed leaves with jagged margins, and clustered flowers (Figure 1). Although native to approximately fifty countries in the Americas and West Africa, it has become widely distributed throughout tropical regions and is recognized as an invasive weed in Thailand and several countries in South-East Asia [8]. Its rapid growth and high biomass production has led *M. capitata* to attract increasing research attention aimed at valorizing weed biomass for non-food industrial applications [9]. Importantly, the species is unsuitable for food or feed use, necessitating alternative utilization pathways. Previous studies have reported various phytoconstituents isolated from *M. capitata*, including flavonoids, coumarins, carbohydrates, glycosides, triterpenes, alkaloids, tannins, and saponins [9,10]. In addition, its allelopathic effects have been previously suggested [8,11], although the specific active compounds and underlying mechanisms remain poorly understood. Its strong competitive ability in natural and agricultural ecosystems suggests that allelopathy may contribute to its invasiveness [9]. However, allelopathic compounds released by plants may affect not only weeds but also crop species. Therefore, evaluating their effects on both target weeds and crops is essential to understand their potential role and limitations in weed management systems. Therefore, the objectives of this study were: (i) to evaluate the allelopathic potential of *M. capitata* leaf extracts collected at different growth stages on selected weed and crop species using germination and seedling growth inhibition bioassays; (ii) to assess its pre-emergence herbicidal activity through residue mulching experiments; (iii) to characterize the chemical profile of the extract through bioassay-guided fractionation and GC-MS analysis; and (iv) to investigate the physiological effects during seed germination. We hypothesized that the allelopathic activity of *M. capitata* varies with plant developmental stage, and that the extract suppresses seed germination by interfering with water uptake and α-amylase activity. Ultimately, this study aims to support sustainable weed management by advancing the development of plant-based natural herbicides that reduce reliance on conventional synthetic agrochemicals.

## 2. Materials and Methods

### 2.1. Plant Materials

*M. capitata* plants were cultivated in sandy loam at the experimental field of King Mongkut’s Institute of Technology Ladkrabang (13.726725° N, 100.780125° E), Bangkok, Thailand, in May 2024, during the rainy season. Leaves were harvested at multiple growth stages: 14, 21, 28, 35, 42, and 49 days after planting (DAP), corresponding approximately to the vegetative leaf development stages (BBCH 12-19) [12]. The harvested leaves were immediately rinsed with running tap water to remove soil and ant debris, then dried in a hot-air oven at 45 °C for 72 h. After drying, the leaves were cut into smaller pieces and pulverized into a fine powder using an electric blender. For the weed bioassay, wild pea (*Phaseolus lathyroides* L.) and barnyardgrass (*Echinochloa crus-galli* [13] Beauv.) were respectively selected as representative broadleaf and grassy weeds. Weed seeds were collected from mature plants from an agricultural field in Phitsanulok province (16.7479° N, 100.1919° E), Thailand. Seeds of maize (*Zea mays* L. var. *saccharata*), rice (*Oryza sativa* L.), cucumber (*Cucumis sativus* L.), cabbage (*Brassica oleracea* L.), and Chinese cabbage (*B. chinensis* Tsen & Lee) were obtained from a local supplier.

### 2.2. Bioassays of Aqueous Extracts from Different Growth Stages of M. capitata on Weed Species

Aqueous extracts were prepared from dried leaves of *M. capitata* collected at selected growth stages (14–49 DAP) by soaking 10 g of powdered leaf material in 90 mL of distilled water at 4 °C for 72 h. The mixtures were then filtered through three layers of cheesecloth followed by Whatman No. 1 filter paper to remove debris. The extracts were freshly prepared and used immediately after filtration for the subsequent bioassays. The inhibitory activities of the aqueous extracts at concentrations of 1.25%, 2.5%, 5%, and 10% (*w*/*v*) on seed germination and seedling growth of barnyardgrass and wild pea were evaluated using Petri dish bioassays. Five milliliters of each treatment solution were added to germination paper placed in 9-cm-diameter glass Petri dishes, and 20 seeds were evenly distributed in each dish. Distilled water was used as the control treatment. Each treatment was replicated four times and arranged in a completely randomized design. The Petri dishes were incubated in a growth chamber at 25/31 °C (night/day) under a 12/12 h dark/light photoperiod and approximately 80% relative humidity. Seed germination was recorded seven days after treatment, and a seed was considered germinated when the radicle protruded beyond the seed coat by at least the length of the seed. Seedling growth was assessed by measuring root and shoot lengths at seven days after treatment. Inhibition percentages of seed germination, root length, and shoot length were calculated using the following equation:

Inhibition (% of control) = 100 − (treatment/control × 100)(1)

### 2.3. Pot Experiment on Mulching with M. capitata Leaf Residues for Pre-Emergence Weed Control

The allelopathic potential of dried leaf residues of *M. capitata* on seed emergence and seedling growth of barnyardgrass and wild pea was evaluated under pot conditions. The soil used in this experiment was sandy loam, prepared by mixing soil and sand at a ratio of 3:1 and filling plastic pots. Twenty seeds of barnyardgrass or wild pea were sown separately in each pot. The dried leaves of *M. capitata* were chopped into small, relatively uniform fragments and applied as surface mulch. Leaf residues were applied at rates of 50, 100, 200, and 400 g/m^2^. Soil without residues (negative control) and leaf residues extracted with ethanol (physical control) were used as the control treatment. Each treatment consisted of four pots arranged in a completely randomized design in the experimental house and watered daily. Seed emergence was recorded at 7 days after treatment. Plant height was measured at 7, 14, 21, and 28 days after treatment, and the dry weight of surviving seedlings was determined at 28 days after treatment. The inhibition percentages of seed emergence, plant height, and dry weight were calculated.

### 2.4. Bioassays of Aqueous Extracts of M. capitata on Selected Crops

To evaluate crop selectivity, aqueous extracts from the growth stage showing the highest inhibitory activity were further tested on seed germination and seedling growth of selected cultivated crops, namely maize, rice, cucumber, cabbage, and Chinese cabbage, using the same Petri dish bioassay procedure described above. The concentration of the *M. capitata* leaf extracts required for 50% growth inhibition of the test plants, defined as the effective concentration (EC_50_), was estimated using the regression equation of the dose–response curve.

### 2.5. Bioassay-Guided Fractionation via Acid-Base Solvent Partitioning

**Figure 2:**
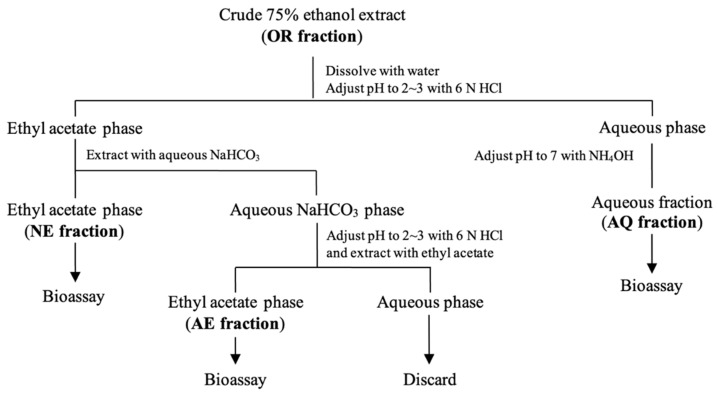
Flow chart for acid–base solvent partitioning of *M. capitata* extracts used in this study.

To identify inhibitory allelochemicals, extracts of *M. capitata* leaves collected at 35 DAP were fractionated using acid–base solvent partitioning following the method described by Laosinwattana et al. [14]. First, dried leaves were extracted with 75% (*v*/*v*) ethanol. The extract was then filtered through Whatman No. 1 filter paper and concentrated to dryness using a rotary evaporator at 45 °C, yielding a sticky residue designated as the original crude extract (OR fraction). The OR fraction was diluted with 0.5 L of distilled water and stirred vigorously on a magnetic stirrer at 45 °C for 20 min to obtain an aqueous solution, which was then acidified to pH 3 using 6 N HCl. The acidified solution was extracted three times with ethyl acetate. The remaining aqueous phase was adjusted to neutral pH using NH_4_OH and evaporated to dryness to obtain the crude aqueous fraction (AQ fraction). The ethyl acetate phase was dried over anhydrous magnesium sulfate (MgSO_4_) and subsequently extracted three times with saturated aqueous sodium bicarbonate (NaHCO_3_). After separation, the ethyl acetate phase was dried over MgSO_4_ and evaporated to obtain the ethyl acetate-soluble neutral fraction (NE fraction). The combined NaHCO_3_ aqueous phases were acidified to neutral pH using HCl and then extracted with ethyl acetate. The resultant extracts were combined, dried over MgSO_4_, and evaporated to obtain the ethyl acetate-soluble acidic fraction (AE fraction). The remaining aqueous phase was discarded (Figure 2). Total phenolic content (TPC) of extracts was determined using the Folin–Ciocalteu colorimetric method following Blainski et al. [15]. In addition, pH and osmotic potential were measured to exclude possible interference effects in the bioassays [16]. Osmotic potential (mbar) was estimated from electrical conductivity (EC; µS/cm) using an empirical conversion factor (Osmotic potential = 0.36 × EC), as described by Mushtaq et al. [17].

Each crude fraction (OR, AQ, NE, and AE) was formulated as a wettable powder (WP). The crude extracts were first dissolved in acetone using a mortar, after which a wettable powder carrier mixture (bentonite: anionic surfactant; 95:5, *w*/*v*) was added at a ratio of 3:7 (*w*/*w*), resulting in formulations containing 30% active ingredient (ai). Stock solutions of each WP formulation were prepared in distilled water at a concentration of 10,000 ppm ai and subsequently diluted to final concentrations ranging from 1250 to 10,000 ppm ai. The inhibitory activities and EC_50_ of the formulated fractions on seed germination and seedling growth of wild pea were evaluated using the same Petri dish bioassay procedure described above. Mover, changes in the morphology of wild pea were observed under a stereo microscope (SZX7, Olympus, Tokyo, Japan) equipped with an EP50 digital microscope camera (CX23, Olympus, Tokyo, Japan) and the EPview software (Olympus, Tokyo, Japan, version 3.7.7).

### 2.6. Physiological Effects of M. capitata Extracts During Seed Germination of Wild Pea

#### 2.6.1. Seed Imbibition

Seed germination and early seedling growth are initiated by water imbibition, which activates embryo development and induces hydrolytic enzymes responsible for mobilizing stored reserves. Among these enzymes, α-amylase plays a critical role by hydrolyzing α-1,4-glucosidic linkages in starch to produce the soluble sugars required for early seedling growth. Therefore, seed imbibition and α-amylase activity were assessed in this study as key physiological processes during seed germination. Water uptake in wild pea seeds exposed to the AE fraction formulated as a WP (1250–10,000 ppm) was measured following the method described by Somala et al. [18]. Distilled water was used as the control. Seed imbibition was assessed at 12, 24, and 48 h after treatment. For each concentration and exposure time, the initial weight (W_1_) of 50 wild pea seeds was recorded, with four replicates per treatment. The seeds were placed on filter paper in 9-cm-diameter Petri dishes and treated with 5 mL of the AE fraction solution. The final seed weight (W_2_) was then recorded for each treatment at the designated time intervals. Seed water uptake was calculated using the following equation:

Seed imbibition (%) = [(W_2_ − W_1_)/W_1_] × 100 (2)

#### 2.6.2. Bioassay Assay of α-Amylase Activity

Following seed imbibition measurement, α-amylase activity was assayed using a modified dinitrosalicylic acid (DNS) method as described by Turk et al. [19]. Briefly, seeds were ground into a fine powder in 4 mL of ice-cold 0.1 M CaCl_2_ solution. The homogenate was centrifuged at 12,000 rpm for 20 min, and the resulting supernatant was used as the enzyme extract. One milliliter of the enzyme extract was incubated with 1 mL of 1% (*w*/*v*) soluble starch prepared in acetate buffer (pH 5.5) at 37 °C for 15 min. The reaction was terminated by adding 1 mL of DNS reagent, followed by heating the mixture in a boiling water bath for 5 min. After cooling under running tap water, absorbance was measured at 560 nm using a Spectronic GENESYS 20 spectrophotometer (Thermo Electron Corporation, Madison, WI, USA). A blank sample containing no enzyme extract was prepared in parallel. A standard curve was constructed using maltose, and α-amylase activity was calculated and expressed as µmol maltose/min/g (FW).

### 2.7. Gas Chromatography-Mass Spectrometry (GC-MS)

The AE fraction from the *M. capitata* OR extract was analyzed using GC-MS analysis. The analysis was performed with a Scion 436 gas chromatograph (SCION Instruments, Livingston, UK) coupled to a Bruker triple quadrupole mass spectrometer (Bruker Daltonics, Bremen, Germany). The operating conditions were as follows: helium flow rate set at 1 mL/min; detection range from 30 to 500 amu; initial oven temperature at 50 °C for 2 min, ramping up to 250 °C at a rate of 20 °C/min, and then held at 250 °C for 18 min. A 1 µL sample was injected in splitless mode using an HP-5MS capillary column (Agilent Technologies, Santa Clara, CA, USA) (30 m length, 0.25 µm film thickness, 0.25 mm ID). The transfer line temperature was maintained at 250 °C, and the ion source temperature was set at 230 °C. Components were tentatively identified by comparing the mass spectra (molecular mass and fragmentation patterns) with the reference library from the National Institute of Standards and Technology (NIST, 2014). No authentic reference standards were analyzed for confirmation. The relative abundances of the components were determined based on the percentage of each peak area relative to the total peak area.

### 2.8. Statistical Analysis

**Figure 3:**
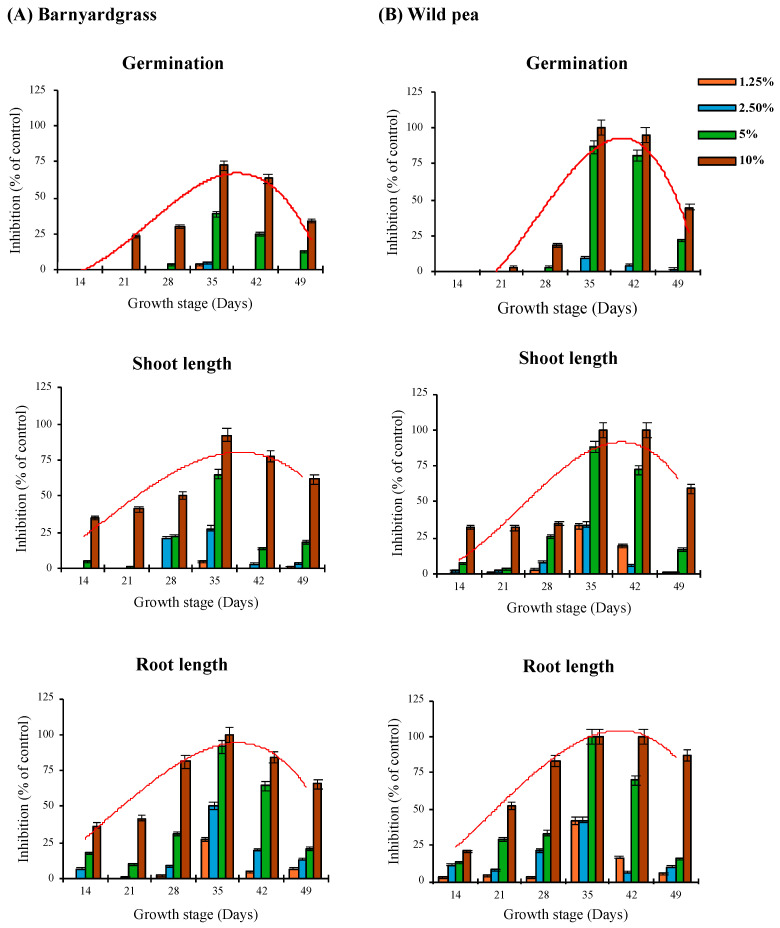
Effect of aqueous extracts from different growth stages of *M. capitata* on seed germination and seedling growth of barnyardgrass (**A**) and wild pea (**B**). The red line represents the polynomial regression trendline. Bars represent the average of four independent biological replicates ± SD (*n* = 4).

All experiments were arranged in a completely randomized design with four replicates per treatment. Seed germination, root length, and shoot length are presented as means ± standard deviation (SD) (*n* = 4). Polynomial regression analysis was used to describe trends in inhibitory activity across plant growth stages, and the fitted curves are shown in Figure 3. Statistical analyses were performed using SAS software version 9.4 (SAS Institute, Cary, NC, USA). Treatment effects were evaluated by one-way ANOVA, followed by Tukey’s HSD test at a significance level of *p* < 0.05. The EC_50_ values and dose–response curves were estimated using R software (version 4.3.2) based on the four-parameter log-logistic model. The model is defined as:

(3) $$y\;=\;c\;+\;\frac{d\;-\;c}{1\;+\;\exp(b(\log x\;-\;\log e))}$$

where y is the response variable, x is the concentration, c and d represent the lower and upper asymptotes, respectively, e corresponds to the EC_50_ value, and b is the slope around the EC_50_. The analysis was performed using the drc package in R.

## 3. Results and Discussion

### 3.1. Allelopathic Activity of M. capitata at Different Growth Stages

Identification of the growth stage with the highest allelopathic activity enables more efficient utilization of allelopathic plants. The results indicated that extracts obtained at 14 DAP did not exhibit inhibitory effects on seed germination of either wild pea or barnyardgrass compared with the control. In contrast, extracts collected at later growth stages (21–49 DAP) exerted varying degrees of inhibitory activity, with the extent of inhibition depending on both growth stage and extract concentration. At 10% (*w*/*v*), germination inhibition (% of control) ranged from 3.75% to 100% in wild pea and from 23.75% to 72.50% in barnyardgrass. Germination of control seeds was consistently above 90% in all experiments. Extracts from the 35 DAP stage exhibited the most pronounced inhibition on both barnyardgrass and wild pea (Figure 3), achieving 72.50% inhibition for barnyardgrass and complete inhibition for wild pea when applied at 10% concentration. This suggests that the allelopathic potential of *M. capitata* peaks at 35 DAP, then declines as the plant ages (see polynomial trendline in Figure 3A,B, red line). In terms of relative inhibitory potency, the various growth stages ranked as follows: 35 > 42 > 49 > 28 > 21 > 14 DAP. When comparing seedling responses to the extracts, germination was the least affected, shoot length and fresh weight exhibited intermediate impacts, and root length showed the greatest negative impact. Tian et al. [20] similarly concluded that root elongation of *Zea mays*, *Glycine max*, and *Triticum aestivum* seedlings is more sensitive to the effects of extracts compared to seed germination. Research on other plant species supports that the composition and optimal concentration of allelochemicals, as well as allelopathic potential, vary with the phenological stage of the donor plant [21,22]. For instance, a study on *Helianthus annuus* found that aqueous extracts collected during the flowering stage suppressed *Lactuca sativa* germination and growth to a greater extent compared to those from the bud formation stage. Similarly, extracts from the full flowering stage of *Brassica* species exhibited significantly higher phytotoxicity compared to extracts from straw material [23]. In several studies, plant material collected during the vegetative stage demonstrated greater allelopathic potential than material from the fruiting stage [22,24]. These findings align with the current study, and collectively indicate that specific growth stages, such as 35 DAP in *M. capitata*, are associated with peak allelopathic activity, likely due to the dynamic nature of allelochemical production during plant development.

### 3.2. Pre-Emergence Herbicidal Activity of M. capitata Leaf Residues

**Figure 4:**
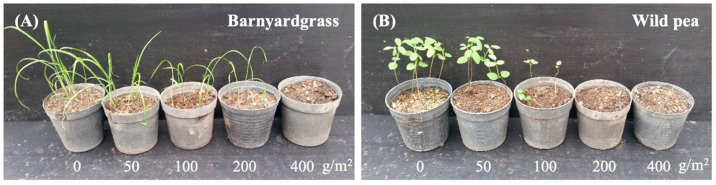
Effect on emergence of barnyardgrass (**A**) and wild pea (**B**) after 28 days of treatment with *M. capitata* leaf residues at different concentrations (50, 100, 200, and 400 g/m^2^).

Application of dried *M. capitata* leaf powder significantly inhibited the emergence and seedling growth of both barnyardgrass and wild pea compared to the control. At the tested application rates, emergence inhibition (% of control) ranged from 42.45% to 100% in wild pea and from 0% to 100% in barnyardgrass. Emergence of the control treatment remained above 90% throughout the experiment. As shown in Table 1, increasing application rates resulted in greater inhibitory effects on weed emergence, plant height, and dry biomass alike. For barnyardgrass, emergence was reduced by 11.25%, 35.00%, and completely inhibited at 100, 200, and 400 g/m^2^, respectively. A similar dose-dependent trend was observed in plant height and dry biomass, reaching complete inhibition at 400 g/m^2^. For wild pea, emergence was more sensitive to the treatment, with 72.50% inhibition at 100 g/m^2^ and complete inhibition at 200 g/m^2^. Plant height inhibition and dry weight reduction also followed a trend. Figure 4 shows that the 28-day assessment revealed that higher mulch doses (200–400 g/m^2^) completely suppressed wild pea emergence, while inducing a gradual decrease in barnyardgrass emergence. The observed inhibitory effect is likely due to allelochemical compounds released from the decomposing leaf residues, which interfered with seed germination and early seedling development. These findings are consistent with previous studies on allelopathic mulching materials. Ravlić et al. [25] reported the incorporation of *H. annuus* residue mulch to effectively suppress *E. colona*, reducing its emergence and growth. Likewise, significant reductions in crop density and total yield of *Cyamopsis tetragonoloba*, *Z. mays*, *Sorghum vulgare*, and *Pennisetum americanum* were observed in fields with *H. annuus* residues [26]. Similarly, Batish et al. [27] observed an herbicidal effect of *Tagetes minuta* leaf powder, application of which at 1, 2, and 4 t/ha significantly inhibited the emergence and biomass accumulation of *E. crus-galli* and *Cyperus rotundus* in rice fields. In another study, vetivergrass leaf residues used as mulch on the soil surface or incorporated into the soil reduced the germination and biomass of *Raphanus sativus* [14]. These findings underscore the potential of using organic leaf powders for enhanced weed management and support their application in integrated weed control strategies.

### 3.3. Effects on Seed Germination and Seedling Growth of Selected Crops

**Figure 5:**
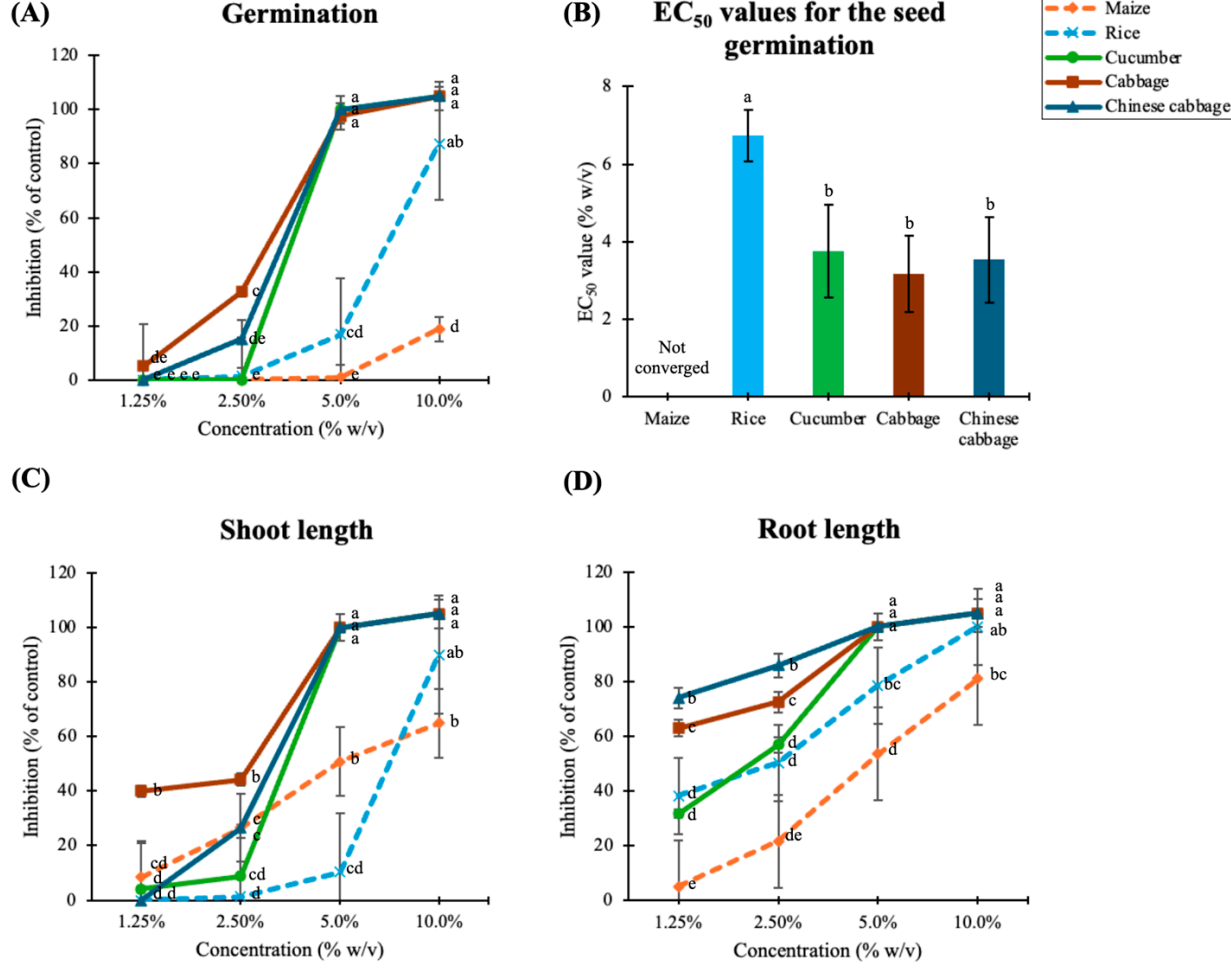
Effects of *M. capitata* aqueous extracts on seed germination (**A**), shoot length (**C**), and root length (**D**) of selected crops, and the EC_50_ values for seed germination (**B**). Values are means ± SD (*n* = 4); different letters in the bars indicate significant differences between the treatments (Tukey’s HSD test; *p* < 0.05). Dashed and solid lines represent monocot and dicot species, respectively.

Aqueous leaf extracts of *M. capitata* suppressed seedling growth of selected cultivated crops, including maize, rice, cucumber, cabbage, and Chinese cabbage, in a concentration-dependent manner (Figure S1). At a concentration of 10% *w*/*v*, seed germination of all tested dicot crops was completely inhibited, whereas germination of maize and rice was reduced to 18.75% and 87.50% of the control, respectively (Figure 5A). The estimated EC_50_ values for seed germination varied among crop species (Figure 5B). The three dicot crops exhibited EC_50_ values ranging from 3.17 to 3.75% *w*/*v*, with no significant differences among them (*p* > 0.05). In contrast, the monocot species exhibited higher EC_50_ values. Rice showed an EC_50_ of 6.74% *w*/*v*, while the EC_50_ for maize exceeded 10% *w*/*v*, indicating greater tolerance to the extract. At the highest tested concentration, seedling growth of all tested crops was reduced to ≤50% of the control (Figure 5C,D). Consistent with the initial weed bioassays, wild pea was more susceptible to *M. capitata* extracts than barnyardgrass. These results suggest that the allelopathic effects of *M. capitata* are species-specific, with dicot species being more sensitive than monocot or grass species. Correspondingly, phytotoxic allelochemicals in *M. capitata* leaf extracts were further isolated by bioassay-guided fractionation and wild pea was used as a representative test species in subsequent experiments.

### 3.4. Bioassay-Guided Fractionation of M. capitata Allelochemicals

Table 2 indicates the pH and osmotic potential of the extracts at 10,000 ppm. The pH values ranged from 4.60 to 5.38, while the osmotic potential varied significantly, with the AQ fraction exhibiting the highest value (1108.80 mbar) and the NE fraction the lowest (0.16 mbar). Previous studies have reported that pH values and osmotic potential within this range do not induce osmotic stress in the germination of various bioassay species [17,28]. Thus, the present results suggest that the observed inhibition of germinability in the test species was primarily due to allelopathic compounds rather than changes in osmotic balance.

**Figure 6:**
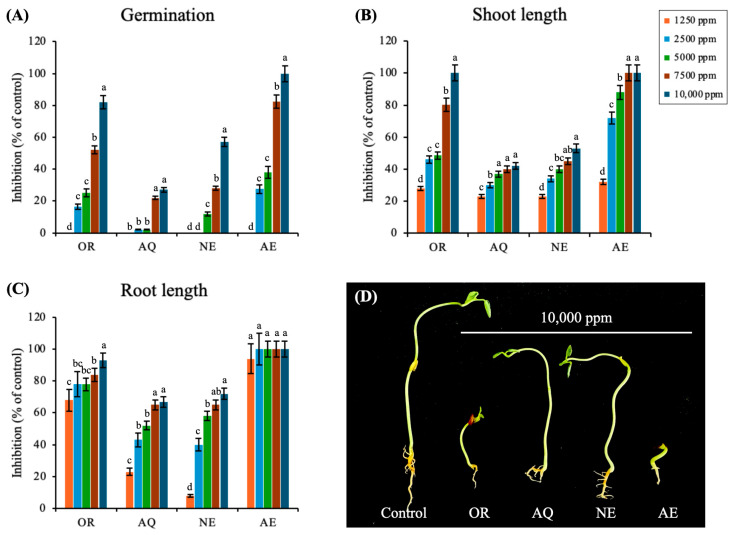
Effects of the ethanol crude extract (OR) and its separated fractions (aqueous, AQ; neutral, NE; and acidic, AE) on seed germination (**A**) and seedling growth (**B**,**C**) in wild pea. Inhibitory effects were measured seven days after treatment (**D**). Bars represent the average of four independent biological replicates ± SD; different letters in the bars indicate significant differences between the treatments at the same extract (Tukey’s HSD test; *p* < 0.05).

Next, the inhibitory effects of the different fractions were compared against the OR fraction (Figure 6A–C). A dose–response analysis further confirmed differences in allelopathic potency among fractions (Figure S2). The AE fraction exhibited the lowest EC_50_ values (Table 2), indicating the highest phytotoxic potency among the tested fractions, whereas the OR, NE, and AQ fractions showed comparatively higher EC_50_ values. The AE fraction completely inhibited wild pea seed germination at 10,000 ppm, whereas the OR, NE, and AQ fractions exhibited respective inhibition rates of 82%, 57%, and 27%. Moreover, as illustrated in Figure 6D, the inhibitory effect of the AE fraction increased relative to the OR, suggesting that phytotoxic compounds were concentrated in this fraction. The results of the TPC analysis (Table 2) further support this conclusion, with the AE fraction exhibiting greater TPC compared to the AQ and NE fractions. The higher phenolic content in the AE fraction aligns with its greater inhibitory activity, reinforcing the role of phenolic compounds in allelopathic interactions. Alsaadawi et al. [29] previously reported phenolic compounds to play crucial roles in phytotoxicity, with significant variations observed among *H. annuus* genotypes such that extracts with higher TPC produced greater weed suppression; this indicates a direct correlation between phenolic concentration and allelopathic potential. In the present study, the enhanced growth inhibition of the AE fraction further indicates potential synergistic action of its components. This is in agreement with Gao and Su [30], who emphasized the importance of phytotoxin mixtures in herbicide development and plant defense mechanisms. In allelopathy and herbicide research, mixture effects are often leveraged to enhance potency, improve efficacy, and reduce selectivity [31]. The strong inhibitory response of the AE fraction in the present study highlights its potential application as a natural herbicide. Accordingly, its chemical composition and mode of action were further investigated.

### 3.5. Gas Chromatography-Mass Spectrometry Analysis of the Acidic Fraction

**Figure 7:**
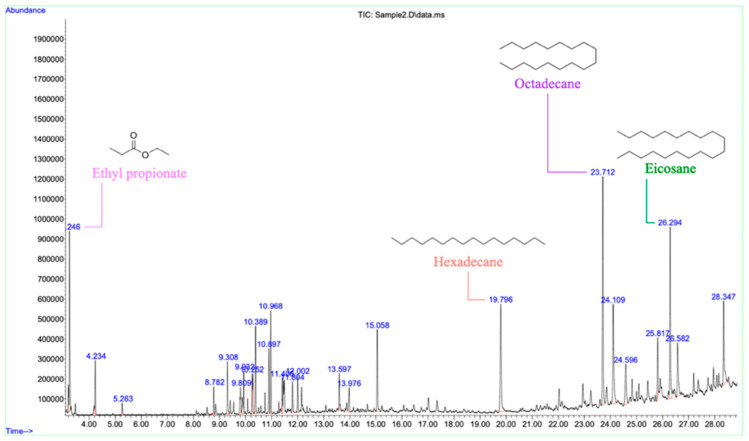
GC-MS chromatogram and chemical structures of the main tentatively identified constituents of the crude AE fraction of *M. capitata*.

In our study, GC-MS analysis of the AE from *M. capitata* tentatively identified 24 constituents, comprising approximately 97.046% of the sample (Table 3 and Figure 7). The major compounds detected in the present study, including long-chain hydrocarbons, fatty acids, and fatty acid esters, are commonly reported as dominant constituents in plant extracts. Similar compounds have also been identified as major constituents in other plant species, such as *Combretum dolichopentalum* [32], *Thymbra capitata* [33], and *Cabomba furcata* [34]. In this study, several long-chain alkanes were tentatively identified, including octadecane (12.447%), hexadecane (9.602%), and eicosane (9.743%). However, these compounds are unlikely to fully account for the observed phytotoxicity in aqueous bioassays due to their low polarity and limited water solubility. Therefore, their specific contributions to the observed phytotoxicity should be further confirmed through bioassays using purified compounds. Nevertheless, previous publications have reported that long-chain hydrocarbons can play roles in plant defense mechanisms by interfering with cell membrane permeability and fluidity in target organisms [35,36]. Aromatic hydrocarbons were tentatively identified, with notable compounds such as 1,2,3,5-tetramethylbenzene (4.351%) and 1-ethyl-2,4-dimethylbenzene (3.929%). These compounds are also often associated with allelopathic activities, potentially inhibiting seed germination and seedling growth of competing species through mechanisms such as disruption of cell division and elongation processes [37,38]. The identification of terpenoids, specifically camphor (1.692%) and (-)-menthol (1.656%), is particularly significant in the context of allelopathy. Terpenoids are well-documented allelochemicals that exhibit a wide range of bioactivities, including inhibition of seed germination, interference with nutrient uptake, and disruption of photosynthetic processes [39]. However, GC-MS primarily detects volatile and semi-volatile compounds and may underrepresent polar or non-volatile allelochemicals. Therefore, the detected compounds should not be directly interpreted as the principal bioactive constituents responsible for the observed phytotoxicity. Overall, the present study provides a preliminary chemical profile of the acidic fraction and serves as a foundation for future targeted investigations. Further bioassay-guided isolation and purified-compound assays are required to determine the specific compounds contributing to the biological activity.

### 3.6. Effect of Acidic Fraction on Water Uptake and α-Amylase Activity

**Figure 8:**
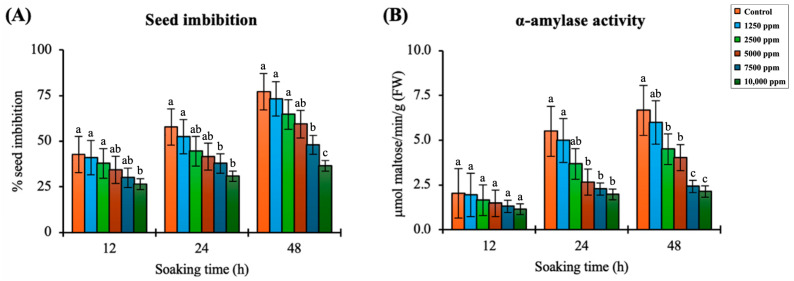
Effects of the AE fraction from *M. capitata* (1250 to 10,000 ppm) on seed imbibition (**A**) and α-amylase activity (**B**) of wild pea seeds over two days of soaking. Bars represent the average of four independent biological replicates ± SD (*n* = 4); different letters in the bars indicate significant differences between the treatments at the same soaking time (Tukey’s HSD test; *p*  <  0.05).

Figure 8A illustrates the effect on water uptake in wild pea seeds of treatment with the AE fraction of *M. capitata* formulated as a WP. In the control treatment, water uptake increased progressively with imbibition time, reaching 42.66%, 57.82%, and 77.06% at 12, 24, and 48 h, respectively. In contrast, seeds treated with 7500 and 10,000 ppm of the AE fraction exhibited significantly lower water uptake at all time points. Although water uptake generally increased with longer imbibition periods, seeds treated with 10,000 ppm AE exhibited only minimal increases over time.

The effects of the AE fraction on α-amylase activity are depicted in Figure 8B. In the control treatment, α-amylase activity increased steadily, reaching 2.03, 5.49, and 6.66 μmol maltose/min/g (FW) at 12, 24, and 48 h, respectively. In contrast, seeds exposed to the AE fraction exhibited markedly lower α-amylase activity, with consistent strong suppression in seeds treated with 7500 and 10,000 ppm AE throughout the incubation period. In the present study, untreated seeds exhibited the highest levels of water uptake and α-amylase activity throughout the experiment, whereas seeds treated with the AE fraction showed significant reductions in both parameters. These results indicate that the allelopathic activity of the AE fraction may suppress seed germination by limiting water uptake and inhibiting α-amylase activity, thereby restricting the availability of energy for early seedling development. This inhibition is consistent with previous studies, such as those by Sooman et al. [40] and Han et al. [41], which reported that plant extracts, including those from black mustard and ginger, can suppress water uptake and α-amylase activity during seed germination. In addition, α-amylase activity in seeds treated with the AE fraction was lower than that in untreated seeds, corroborating the findings of Kato-Noguchi and Macías [42], who demonstrated that 6-methoxy-2-benzoxazolinone derived from Gramineae species inhibits α-amylase activity in lettuce seeds.

## 4. Conclusions

This study demonstrates that the allelopathic activity of the invasive weed *M. capitata* is influenced by plant growth stage, resulting in differential phytotoxic effects. The greatest inhibitory activity was observed in leaves collected at 35 days after planting. The allelopathic effects were species-specific, with dicot species showing greater sensitivity than monocot species, indicating selective phytotoxicity. Bioassay-guided fractionation revealed the ethyl acetate-soluble acidic fraction to exhibit the highest inhibitory activity. Further physiological investigations showed this fraction to significantly reduce water uptake and suppress α-amylase activity during seed germination. Overall, these findings highlight the allelopathic potential of *M. capitata*. Valorization of this invasive weed may contribute to sustainable weed management strategies. Further studies should evaluate the herbicidal activity of *M. capitata* against a broader range of weed species.

## Figures and Tables

**Table 1:** Inhibition of seed emergence, plant height, and plant dry weight of barnyardgrass and wild pea following treatment with *M. capitata* leaf residue mulch in a pot test.

Application Rate	Inhibition (% of Control)					
	Plant Height (Days After Treatment)				Emergence	Dry Weight
	7	14	21	28		
Barnyardgrass						
50 g/m^2^	0.00 d	0.00 d	0.00 d	0.00 d	0.00 d	0.00 d
100 g/m^2^	38.50 c	30.00 c	36.75 c	35.00 c	11.25 c	33.58 c
200 g/m^2^	40.50 b	40.25 b	45.00 b	49.75 b	35.00 b	51.87 b
400 g/m^2^	100.00 a	100.00 a	100.00 a	100.00 a	100.00 a	100.00 a
Wild pea						
50 g/m^2^	13.75 c	10.00 c	8.75 c	3.50 c	42.75 c	46.49 c
100 g/m^2^	35.25 b	53.50 b	43.50 b	42.25 b	72.50 b	70.57 b
200 g/m^2^	100.00 a	100.00 a	100.00 a	100.00 a	100.00 a	100.00 a
400 g/m^2^	100.00 a	100.00 a	100.00 a	100.00 a	100.00 a	100.00 a

In each column, means having the same letter are not significantly different by Tukey’s HSD test (*p* < 0.05).

**Table 2:** pH, total phenolic content (TPC), electrical conductivity, and osmotic potential of *M. capitata* extract at 10,000 ppm, and the EC_50_ value of each crude extract on wild pea seedling growth.

Extract	TPC ^a^	pH	EC (μS/cm)	OS (mbar)	EC_50_ Values for Wild Pea Seedling Growth (ppm)		
					Germination	Shoot Length	Root Length
OR	1613 a	5.38 b	139.4 b	50.18 b	7314.8 b	5112.8 b	919.1 b
AQ fraction	1278 b	6.03 a	3080 a	1108.80 a	Not converged	Not converged	4444.4 a
NE fraction	1220 b	6.33 a	0.45 c	0.16 c	9396.6 a	9062.5 a	3888.9 a
AE fraction	1620 a	4.60 c	0.70 c	0.25 c	5674.2 c	1812.5 c	664.9 b

^a^ TPC expressed as mg GAE/100 g crude extract. In each column, means having the same letter are not significantly different by Tukey’s HSD test (*p* < 0.05).

**Table 3:** Chemical constituents of *M. capitata* crude AE fraction identified by GC-MS.

Constituent	R.T. ^a^	% ^b^	Formula	Compound Nature
Ethyl propionate	3.246	7.391	C_5_H_10_O_2_	Ester
Toluene	4.234	2.374	C_7_H_8_	Aromatic hydrocarbon
1-deoxy-D-mannitol	5.263	0.614	C_6_H_13_NO_7_	Polyol
1,2,4-trimethyl benzene	8.781	1.270	C_9_H_12_	Aromatic hydrocarbon
Mesitylene	9.308	2.522	C_9_H_12_	Aromatic hydrocarbon
1-methyl-3-propylbenzene	9.809	1.923	C_10_H_14_	Aromatic hydrocarbon
2-ethyl-1,4-dimethylbenzene	9.932	1.835	C_10_H_14_	Aromatic hydrocarbon
1-ethyl-3,5-dimethylbenzene	10.252	1.703	C_10_H_14_	Aromatic hydrocarbon
1-ethyl-2,4-dimethylbenzene	10.389	3.929	C_10_H_14_	Aromatic hydrocarbon
1,2,3,5-tetramethylbenzene	10.968	4.351	C_10_H_14_	Aromatic hydrocarbon
Camphor	11.406	1.692	C_10_H_16_O	Terpenoid
(-)-menthol	11.804	1.656	C_10_H_16_O	Terpenoid
Naphthalene	12.002	1.720	C_10_H_8_	Aromatic hydrocarbon
*cis*-2-tert-butylcyclohexyl acetate	13.597	1.402	C_12_H_22_O_2_	Ester
2,6,10-trimethyltetradecane	13.976	1.396	C_17_H_36_	Alkane
Tetradecane	15.058	4.526	C_14_H_30_	Alkane
Hexadecane	19.796	9.602	C_16_H_34_	Alkane
Octadecane	23.712	12.447	C_18_H_38_	Alkane
Isopropyl myristate	24.109	7.503	C_17_H_34_O_2_	Fatty acid ester
Versalide	24.596	4.052	C_18_H_26_O	Aromatic hydrocarbon
Caproic acid	25.817	3.143	C_6_H_12_O_2_	Fatty acid
Eicosane	26.294	9.743	C_20_H_42_	Alkane
Isopropyl palmitate	26.582	4.309	C_19_H_38_O_2_	Fatty acid ester
Heptacosane	28.347	5.943	C_27_H_56_	Alkane
Total identified		97.046		

^a^ Retention time (min); ^b^ Relative area percentage (peak area relative to the total peak area, %).

## Data Availability

The original contributions presented in this study are included in the article. Further inquiries can be directed to the corresponding authors.

## Supplementary Materials

The following supporting information can be downloaded at: https://www.mdpi.com/article/10.3390/plants15050832/s1; Figure S1. Dose–response curves of *M. capitata* aqueous leaf extracts on seed germination (A) and seedling growth (B and C) of selected crops. Dose–response relationships were modeled using a four-parameter log-logistic model. Points represent mean inhibition values (% of control) from four independent biological replicates (*n* = 4), and solid lines indicate fitted curves across the tested concentration range (1.25–10% *w*/*v*). Concentrations are presented on a logarithmic scale. Figure S2. Dose–response curves of the ethanol crude extract (OR) and its separated fractions (aqueous, AQ; neutral, NE; and acidic, AE) on seed germination (A) and seedling growth (B and C) in wild pea. Dose–response relationships were modeled using a four-parameter log-logistic model. Points represent mean inhibition values (% of control) from four independent biological replicates (*n* = 4), and solid lines indicate fitted curves across the tested concentration range (1250–10,000 ppm). Concentrations are presented on a logarithmic scale.

## References

[1] Khan I., Rehman O., Khan S., Alsamadany H., Alzahrani Y. (2020). Effect of different herbicides, plant extracts and mulches on yield and yield components of maize. Planta Daninha.

[2] Nath C.P., Singh R.G., Choudhary V.K., Datta D., Nandan R., Singh S.S. (2024). Challenges and alternatives of herbicide-based weed management. Agronomy.

[3] Inderjit, Duke S.O. (2003). Ecophysiological aspects of allelopathy. Planta.

[4] Farooq N., Abbas T., Tanveer A., Jabran K., Mérillon J.M., Ramawat K. (2020). Allelopathy for weed management. Co-Evolution of Secondary Metabolites.

[5] Somala N., Manichart N., Thongbang M., Wichittrakarn P., Laosinwattana C., Teerarak M. (2025). Characterization of *Diaporthe* fungal extract composition and phytotoxicity on the aquatic noxious weed *Eichhornia crassipes*: Inhibitory effects on photosynthetic machinery and membrane integrity. Asian J. Agric. Biol..

[6] Xu Y., Chen X., Ding L., Kong C.-H. (2023). Allelopathy and allelochemicals in grasslands and forests. Forests.

[7] Zhang Z., Liu Y., Yuan L., Weber E., van Kleunen M. (2021). Effect of allelopathy on plant performance: A meta-analysis. Ecol. Lett..

[8] Vaishali R., Alka C. (2019). Allelopathic effect of some plants on morphological attributes of invasive alien weed: *Malachra capitata* L.. GSC Biol. Pharm. Sci..

[9] Cervantes-Ceballos L., Sánchez-Hoyos J., Sanchez-Hoyos F., Torres-Niño E., Mercado-Camargo J., Echeverry-Gómez A., Jotty Arroyo K., del Olmo-Fernández E., Gómez-Estrada H. (2022). An overview of genus *Malachra* L.-Ethnobotany, phytochemistry, and pharmacological activity. Plants.

[10] Pratyusha S., Jayasri P., Elumalai A. (2012). Study on phytochemical profile and antiulcerogenic effect of *Malachra capitata* in albino Wistar rats. Int. J. Preclin. Pharm. Res..

[11] Joshi N., Nangia N., Joshi A. (2015). Seed germination studies on allelopathic effects of weeds on *Vigna radiata* L.. Int. J. Bioassays.

[12] Niemenak N., Cilas C., Rohsius C., Bleiholder H., Meier U., Lieberei R. (2010). Phenological growth stages of cacao plants (*Theobroma* sp.): Codification and description according to the BBCH scale. Ann. Appl. Biol..

[13] Del Prado-Audelo M.L., Cortés H., Caballero-Florán I.H., González-Torres M., Escutia-Guadarrama L., Bernal-Chávez S.A., Giraldo-Gomez D.M., Magaña J.J., Leyva-Gómez G. (2021). Therapeutic applications of terpenes on inflammatory diseases. Front. Pharmacol..

[14] Laosinwattana C., Phuwiwat W., Charoenying P. (2007). Assessment of allelopathic potential of vetivergrass (*Vetiveria* spp.) ecotypes. Allelopath. J..

[15] Blainski A., Lopes G.C., De Mello J.C.P. (2013). Application and analysis of the Folin Ciocalteu method for the determination of the total phenolic content from *Limonium brasiliense* L.. Molecules.

[16] Wichittrakarn P., Manichart N., Laosinwattana C., Charoenying P., Sikhao P., Passara H. (2025). Development of a *Melia azedarach* L. based natural herbicide: Formulation and efficacy studies. J. Agric. Food Res..

[17] Somala N., Manichart N., Laosinwattana C., Wichittrakarn P., Yoneyama K., Teerarak M., Chotsaeng N. (2024). Oxidative damage in *Echinochloa crus−galli* seeds exposed to *Diaporthe* sp. (Diaporthales, Ascomycota) fungal extract during germination. Front. Agron..

[18] Turk M., Shatnawi M., Tawaha A. (2003). Inhibitory effects of aqueous extracts of black mustard on germination and growth of alfalfa. Weed Biol. Manag..

[19] Tian M., Li Q., Zhao W., Qiao B., Shi S., Yu M., Li X., Li C., Zhao C. (2022). Potential allelopathic interference of *Abutilon theophrasti* Medik. powder/extract on seed germination, seedling growth and root system activity of maize, wheat and soybean. Agronomy.

[20] Zribi I., Omezzine F., Haouala R. (2014). Variation in phytochemical constituents and allelopathic potential of *Nigella sativa* with developmental stages. S. Afr. J. Bot..

[21] de Sousa M.V., de Farias S.G.G., de Castro D.P., e Silva R.B., de Oliveira Silva D.Y.B., Dias B.A.S., da Silva A.F., dos Santos G.N.L., de Matos D.C.P., de Almada Oliveira C.V. (2018). Allelopathy of the leaf extract of eucalyptus genetic material on the physiological performance of millet seeds. Am. J. Plant Sci..

[22] Jafariehyazdi E., Javidfar F. (2011). Comparison of allelopathic effects of some brassica species in two growth stages on germination and growth of sunflower. Plant Soil Environ..

[23] Omezzine F., Haouala R. (2013). Effect of *Trigonella foenum-graecum* L. development stages on some phytochemicals content and allelopathic potential. Sci. Hortic..

[24] Ravlić M., Markulj Kulundžić A., Baličević R., Marković M., Viljevac Vuletić M., Kranjac D., Sarajlić A. (2022). Allelopathic potential of sunflower genotypes at different growth stages on lettuce. Appl. Sci..

[25] Batish D., Tung P., Singh H., Kohli R. (2002). Phytotoxicity of sunflower residues against some summer season crops. J. Agron. Crop Sci..

[26] Batish D.R., Arora K., Singh H.P., Kohli R.K. (2007). Potential utilization of dried powder of *Tagetes minuta* as a natural herbicide for managing rice weeds. Crop Prot..

[27] Mushtaq W., Ain Q., Siddiqui M., Hakeem K.R. (2019). Cytotoxic allelochemicals induce ultrastructural modifications in *Cassia tora* L. and mitotic changes in *Allium cepa* L.: A weed versus weed allelopathy approach. Protoplasma.

[28] Wu L.-M., Fang Y., Yang H.-N., Bai L.-Y. (2019). Effects of drought-stress on seed germination and growth physiology of quinclorac-resistant *Echinochloa crusgalli*. PLoS ONE.

[29] Alsaadawi I.S., Sarbout A.K., Al-Shamma L.M. (2012). Differential allelopathic potential of sunflower (*Helianthus annuus* L.) genotypes on weeds and wheat (*Triticum aestivum* L.) crop. Arch. Agron. Soil Sci..

[30] Gao W.-T., Su W.-H. (2024). Weed management methods for herbaceous field crops: A review. Agronomy.

[31] Vajja N.R., Meinke H., Kropff M.J., Anten N.P., Whitbread A.M., Kumar U., Parsons D. (2025). Incorporating knowledge of allelopathic interactions can improve productivity and sustainability of crop rotations in the semi-arid tropics. J. Agric. Food Res..

[32] Sharma S., Kumar R. (2018). Influence of harvesting stage and distillation time of damask rose (*Rosa damascena* Mill.) flowers on essential oil content and composition in the Western Himalayas. J. Essent. Oil Bear. Plants.

[33] Khedhri S., Polito F., Caputo L., Manna F., Khammassi M., Hamrouni L., Amri I., Nazzaro F., De Feo V., Fratianni F. (2022). Chemical composition, phytotoxic and antibiofilm activity of seven eucalyptus species from Tunisia. Molecules.

[34] Khedhri S., Polito F., Caputo L., Khammassi M., Dhaouadi F., Amri I., Hamrouni L., Mabrouk Y., Fratianni F., Nazzaro F. (2024). Antimicrobial, herbicidal and pesticidal potential of *Tunisian eucalyptus* species: Chemoprofiling and biological evaluation. Heliyon.

[35] Wang H., Lin W., Zhang D., Yang R., Zhou W., Qi Z. (2022). Phytotoxicity of chemical compounds from *Cinnamomum camphora* pruning waste in germination and plant cultivation. Int. J. Environ. Res. Public Health.

[36] Sharma P., Kumar S. (2021). Characterization and phytotoxicity assessment of organic pollutants in old and fresh municipal solid wastes at open dump site: A case study. Environ. Technol. Innov..

[37] Ujowundu F.N. (2017). In vitro evaluation of free radical-scavenging potentials of ethanol extract of *Combretum dolichopentalum* leaves. Glob. Drugs Ther..

[38] Saoulajan C., Boujida N., El Mihyaoui A., El Baakili A., Alshahrani M.M., Lee L.-H., Bouyahya A. (2022). Phytochemistry, pharmacological investigations, industrial applications, and encapsulation of *Thymbra capitata* L., a review. Trends Food Sci. Technol..

[39] Shefeek S., Jaiganesh K. (2020). GC-MS analysis of ethanolic extract of *Cabomba furcata* Schult. & Schult. F: An aquatic plant. J. Pharmacogn. Phytochem..

[40] Sooman M.I., Odat N., Abu-Romman S., Hasan M., Al-Tawaha A.R. (2020). The effects of allelopathy of mustard (*Brassica nigra*) on water content and gene expression of cultivated barley (*Hordeum vulgare*). Res. Crops.

[41] Han C.-M., Pan K.-W., Wu N., Wang J.-C., Li W. (2008). Allelopathic effect of ginger on seed germination and seedling growth of soybean and chive. Sci. Hortic..

[42] Kato-Noguchi H., Macías F.A. (2005). Effects of 6-methoxy-2-benzoxazolinone on the germination and α-amylase activity in lettuce seeds. J. Plant Physiol..

