# Supplementary material for: Herbicidal Activity of the Invasive Weed Malachra capitata L.: Growth Stage Dependence, Bioassay-Guided Fractionation, and Physiological Effects on Seed Germination
Source: Plants (Basel). 2026 Mar 8;15(5):832. doi: 10.3390/plants15050832 (PMC12987214; doi:10.3390/plants15050832)
Supplement: Supplementary file 1 [file plants-15-00832-s001.zip › plants-4167176-supplementary.pdf]

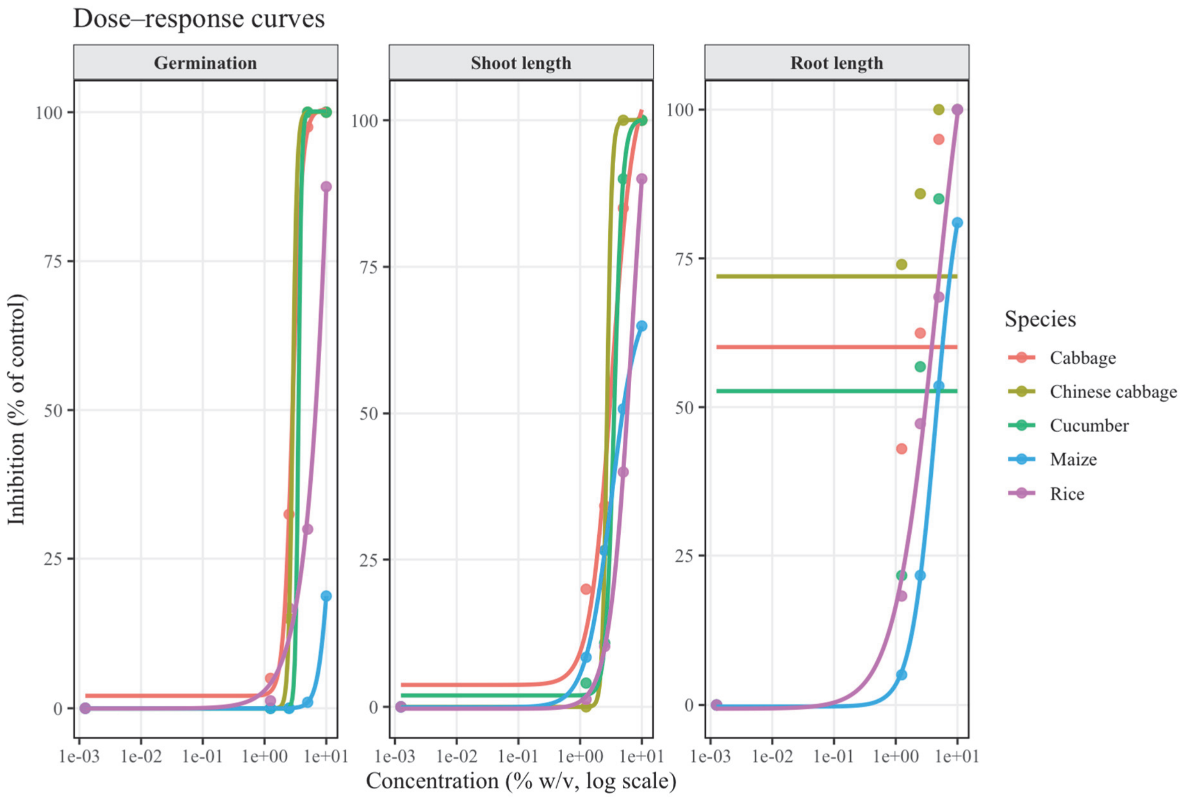

Figure S1. Dose–response curves of *M. capitata* aqueous leaf extracts on seed germination (A) and seedling growth (B and C) of selected crops. Dose–response relationships were modeled using a four-parameter log-logistic model. Points represent mean inhibition values (% of control) from four independent biological replicates (n = 4), and solid lines indicate fitted curves across the tested concentration range (1.25–10% *w*/*v*). Concentrations are presented on a logarithmic scale.

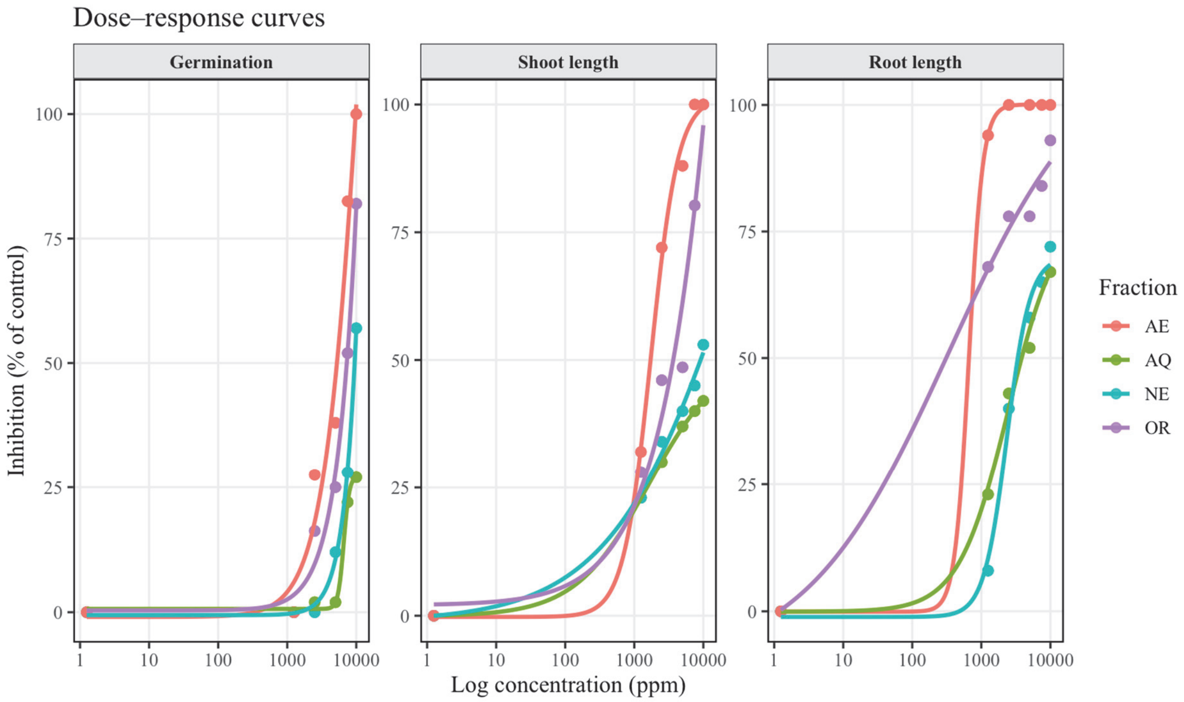

Figure S2. Dose–response curves of the ethanol crude extract (OR) and its separated fractions (aqueous, AQ; neutral, NE; and acidic, AE) on seed germination (A) and seedling growth (B and C) in wild pea. Dose–response relationships were modeled using a four-parameter log-logistic model. Points represent mean inhibition values (% of control) from four independent biological replicates (n = 4), and solid lines indicate fitted curves across the tested concentration range (1250–10,000 ppm). Concentrations are presented on a logarithmic scale.
